# Abnormal topological organization in white matter structural networks in survivors of acute lymphoblastic leukaemia with chemotherapy treatment

**DOI:** 10.18632/oncotarget.19104

**Published:** 2017-07-08

**Authors:** Liwei Zou, Lianzi Su, Rongmiao Qi, Fang Bao, Xianjing Fang, Longsheng Wang, Zhimin Zhai, Dan Li, Suisheng Zheng

**Affiliations:** ^1^ Department of Radiology, The Second Hospital of Anhui Medical University, Anhui, China; ^2^ Department of Hematology, The Second Hospital of Anhui Medical University, Anhui, China; ^3^ Department of Scientific Research, The Second Hospital of Anhui Medical University, Anhui, China; ^4^ Medical Image Research Center, Anhui Medical University, Anhui, China

**Keywords:** acute lymphoblastic leukaemia, chemotherapy, network, white matter, diffusion tensor imaging

## Abstract

Previous diffusion tensor imaging (DTI) studies have detected white matter (WM) integrity abnormalities in some specific fibre bundles in acute lymphoblastic leukaemia (ALL) patients with chemotherapy. However, little is known about the changes in the topological organization of the WM structural network in ALL patients with chemotherapy. In the present study, we acquired DTI datasets from 28 ALL patients (mean age: 40.71 ± 8.58 years, years since diagnosis: 7–38) with chemotherapy and 20 matched healthy controls (mean age: 42.95 ± 6.39 years) and performed WM network analysis using a deterministic fibre-tracking approach. Graph theoretical analysis was used to compare the topological parameters of the WM networks between the two groups. Both ALL patients with chemotherapy and healthy controls had small-worldness in their WM networks. ALL patients showed significantly reduced global network efficiency, as indicated by the abnormally decreased clustering coefficient Cp and the normalized clustering coefficient γ and increased shortest path length Lp compared with healthy controls. Moreover, hubs were located more in parietal regions of healthy controls and in temporal regions in the ALL patients. We revealed the abnormal topological organization of the WM networks of ALL patients with chemotherapy, which may improve our understanding of the neural mechanism of chemotherapy in ALL from a WM topological organization level.

## INTRODUCTION

Acute lymphoblastic leukaemia (ALL) is the most common cancer among children, accounting for 74% of all leukaemias and 18% of all cancers [1, 2]. ALL survivors suffer from long-term neurocognitive impairment and poor health-related quality of life (QoL) [3]. The historical treatment of central nervous system (CNS)-directed therapy has resulted in a 5-year event-free survival rate of approximately 80% [4]. CNS-directed therapies mainly consist of cranial irradiation, chemotherapy, and combinations of these two methods. Among these CNS-directed therapies, cranial irradiation has been implicated as the cause of neurocognitive impairments [5, 6]. However, neurocognitive outcomes after chemotherapy treatment without cranial irradiation are inconsistent. Candidate mechanisms for cognitive impairment include direct neurotoxic effects causing atrophy of grey matter (GM) and/or demyelination of the white matter (WM), secondary immunologic responses leading to inflammatory reactions, microvascular damage, and genetic vulnerabilities [7–9].

In the past decades, the advent of neuroimaging techniques has provided insights into the anatomic substrates of potentially toxic treatment effects. Studies on brain structural and functional alterations (including WM integrity [10–12], GM volume [13–15], resting-state magnetic resonance imaging (MRI) [16], task-state MRI [17–19], and cortical thickness [20, 21]) related to ALL patients who take chemotherapy treatment has been accumulating in recent years. However, these studies have only focussed on regional brain changes, and little is known about global topological alterations specific to ALL with chemotherapy.

Currently, growing amounts of brain network studies have aimed to reconstruct whole-brain WM connectivity with diffusion tensor imaging (DTI) tractography based on graph theory [22, 23]. Graph theoretical analysis provides a powerful tool for quantifying the organization of network connectivity by defining a graph as a set of nodes (brain regions) and edges (structural connections) [23–25]. These methods have revealed many important topological properties, such as small-world, which is characterized by a high global integration and a high local specialization between brain regions [26–30], and the network efficiency that characterizes the fault tolerance of the network [31]. However, no studies have reported ALL with chemotherapy changes in WM brain networks or have investigated the topological differences between ALL patients and healthy controls.

In the present study, we used DTI tractography and graph theory to construct the WM structural networks in ALL patients with chemotherapy and healthy controls. Then, small-world properties and global parameters were compared between these two groups. We hypothesized that the network topographical structure might have been disrupted in ALL patients and hope to provide new insights into the mechanisms of chemotherapy-induced brain impairment.

## RESULTS

### Demographics

There were no significant differences in terms of age (two sample *t*-test, *t* = 1.433, *p* = 0.159) and gender (chi-square test, χ^2^ = 1.067, *p* = 0.302) between the ALL patients and healthy controls (Table 1).

**Table 1:** Demographic characteristics of study participants

Items	ALL patients	Healthy controls	*P*-value
N	28	20	–
Age (year)	40.71 ± 8.58	42.95 ± 6.39	0.33^a^
Gender (M/F)	14/14	8/12	0.49^b^

Note: a *p* value from *t* test, b *p* value from chi-square test.

### Small-worldness

**Figure 1:**
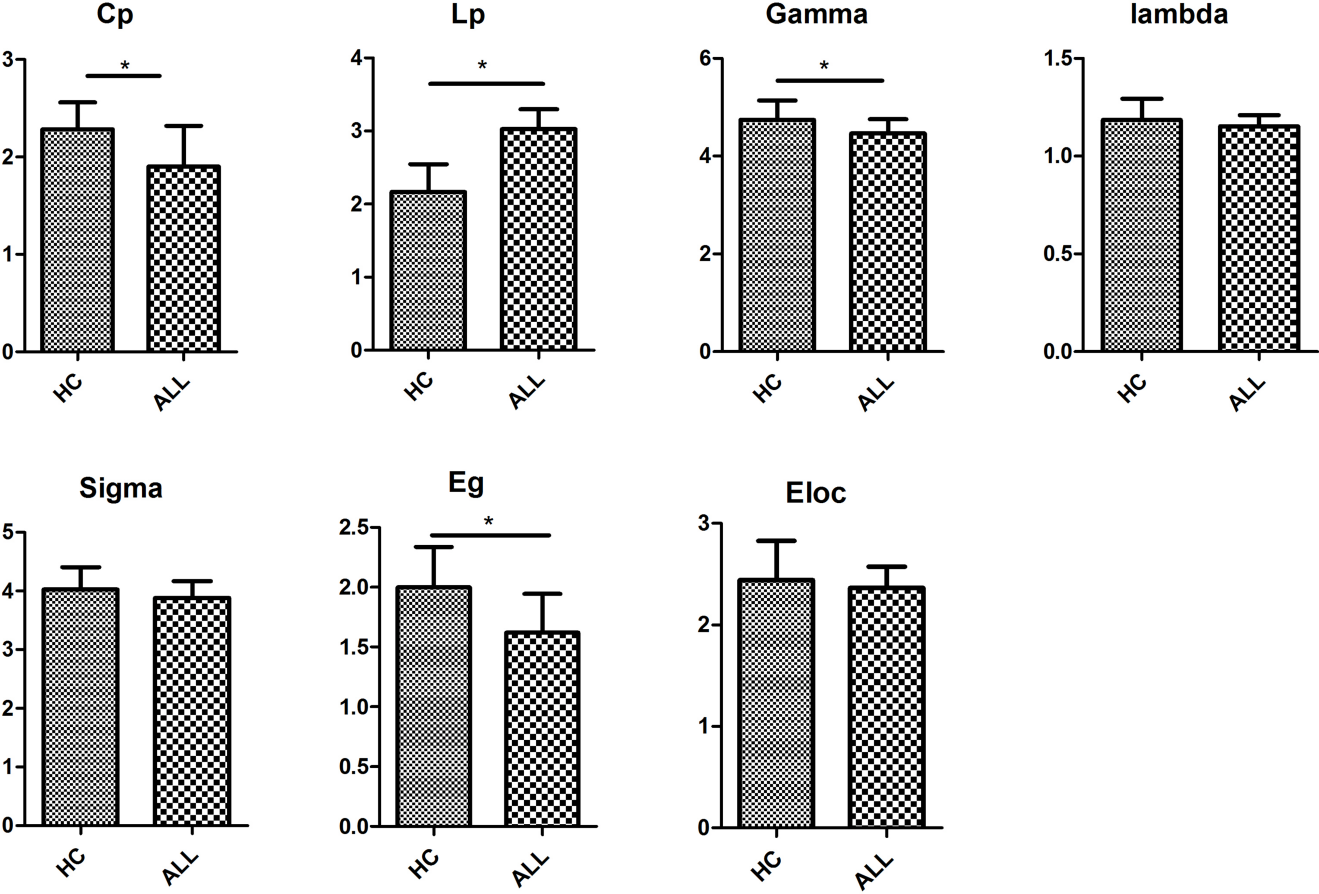
Differences in global metrics of the structural networks between ALL and HC groups Error bar represent standard errors. Cp, clustering coefficient; Lp, shortest path length; Gamma, normalized clustering coefficient; Lambda, normalized shortest path length; sigma, small-worldness; E_g_, global efficiency; E_loc_, local efficiency. ALL, acute lymphoblastic leukemia with chemotherapy treatment, HC, healthy controls.

At the threshold of three fibres, the structural networks of the ALL patients and healthy controls had higher normalized clustering coefficients (γ_ALL_ = 4.462 ± 0.055, γ_HC_ = 4.744 ± 0.088) but almost identical normalized shortest path lengths (λ_ALL_ = 1.152 ± 0.011, λ_HC_=1.185 ± 0.024) relative to matched random networks, which suggested that the two groups exhibited typical small-world organizations in their structural networks. Statistical analysis indicated that ALL patients had a significant increase in the shortest path length Lp (*P* < 0.01) and significant decreases in the clustering coefficient C_p_ (*P* < 0.01) and the normalized clustering coefficients γ (*P* < 0.01). No group differences were found for normalized shortest path length λ and small-world σ (Figure 1 and Supplementary Table 1).

### Network efficiency

We found significantly lower global efficiency Eg (*P* < 0.01) for ALL patients relative to healthy controls (Figure 1). No group difference was found for local efficiency Eloc (Figure 1).

### Hubs

**Figure 2:**
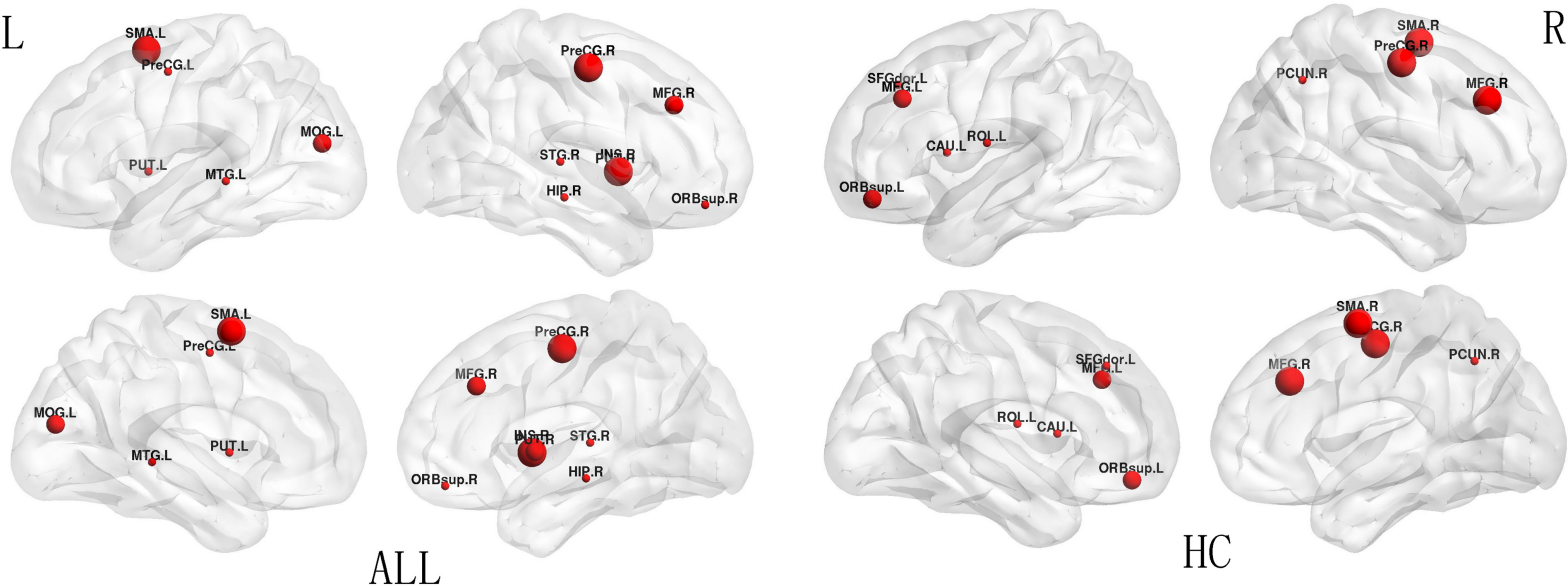
Hub distribution between ALL and HC groups The size of nodes indicates the betweenness centrality (BC). PreCG, precentral gyrus; MFG, middle frontal gyrus; SMA, supplementary motor area; INS, insula; MOG, middle occipital gyrus; PUT, putamen; SFGorb, superior frontal gyrus_orbital; HIP, hippocampus; STG, superior temporal gyrus; MTG, middle temporal gyrus; SFGdor, superior frontal gyrus_ dorsolateral; ROL, rolandic operculum; PCUN, precuncus; CAU, caudate nucleus; R, right; L, left.

The regions were defined as network hubs if their nodal betweenness centrality was one standard deviation (SD) greater than the average of the network. The study identified 9 hub nodes of the WM structural networks in the healthy control group and 12 hub nodes in the ALL group. In both groups, 2 brain regions were identified as hubs in common, including the right precentral gyrus (PreCG.R) and the right middle frontal gyrus (MFG.R). In addition, some brain regions, including the left supplementary motor area (SMA.L), the right insula (INS.R), the left middle occipital gyrus (MOG.L), the bilateral putamen (PUT), the left precentral gyrus (PreCG.L), the right superior frontal gyrus_orbital (SFGorb.R), the right hippocampus (HIP.R), the right superior temporal gyrus (STG.R), and the left middle temporal gyrus (MTG.L) were identified as hubs in the ALL group but not in the healthy control group. Seven brain regions, including the right supplementary motor area (SMA.R), the left superior frontal gyrus_orbital (SFGorb.L), the left middle frontal gyrus (MFG.L), the left superior frontal gyrus_dorsolateral (SFGdor.L), the left rolandic operculum (ROL.L), the right precuneus (PCUN.R), and the left caudate nucleus (CAU.L), were identified as hubs in the healthy controls group but not in the ALL group (Figure 2).

## DISCUSSION

In the present study, we explored whether there were aberrant topological properties in WM networks in ALL patients with chemotherapy treatment and healthy controls. Our main findings were as follows: a) small-world organizations were present in both ALL patients with chemotherapy and controls, which is agreement with a previous study [42]; b) increased shortest path length Lp (*P* < 0.01), decreased in clustering coefficient C_p_ (*P* < 0.01), and decreased normalized clustering coefficients γ (*P* < 0.01) were present in ALL patients; c) lower global efficiency Eg (*P* < 0.01) was present in ALL patients; d) hubs were located more in the parietal regions of healthy controls and in the temporal regions in ALL patients; and e) alterations in nodal betweenness centrality were present in both groups.

Our study is the first to find that the ALL patients with chemotherapy treatment demonstrated reduced global connectivity in WM structure compared with healthy controls. This global hypo-connectivity in ALL patients with chemotherapy treatment is reflected by two abnormal indexes for small world measures (decreased γ and increased λ). As expected, the significantly decreased γ in ALL patients compared with healthy controls has been suggested to reduce global connectivity efficiency [23], which is in line with a previous study [42]. Global efficiency is known to indicate the capacity to transfer information between different nodes of the brain network and is a comprehensive index for parallel information processing capabilities [39, 43]. Brain networks with small-world σ confer resilience against pathological attack, and impairments of these properties are caused by chemotherapy treatment in ALL patients.

We demonstrated a lower network efficiency in ALL patients with chemotherapy treatment compared with healthy controls, which suggested a less optimal organization for ALL patients. The significantly decreased C_p_ and increased L_p_ in ALL patients indicate the loss of the capacity to transmit specialized information rapidly among distant brain regions. It is suggested that the structural aberrations in some local brain areas might have contributed to the reduced clustering and longer path length in ALL patients compared with healthy controls.

There have been several studies on the morphological analysis of ALL patients with chemotherapy treatment [15, 20, 21, 44], but the results have been inconsistent. Reduced thickness in the right precentral gyrus [21], decreased GM volume in several brain regions [15, 20], and increased GM volume in the caudate [44] have also been reported. A possible explanation for these findings might be the mixing of various aetiologies, or the analyses were performed using different methods. By employing graph theoretical analysis for structural imaging, our study revealed the different structural connectivity networks between ALL patients with chemotherapy treatment and healthy controls.

The present study found that the hubs were mostly located in the parietal and temporal regions, which is compatible with former findings in which hubs were primarily located in parietal areas [41], reflecting the main functions associated with these areas [23]. However, there was little overlap distribution of hubs between the two groups. Specifically, hubs in parietal regions accounted for a great proportion of all of the hubs in healthy controls, while more hubs were found in temporal regions in ALL patients with chemotherapy treatment. These findings suggest that parietal regions are more important in healthy populations, while temporal regions are more important in ALL patients.

A number of limitations need to be noted regarding the current study. First, the methodology of deterministic tractography to reconstruct structural connectivity cannot accurately map out all the fibres and has the limitation of tracking crossing fibres. Second, the sample size is limited, and the results need to be replicated in large samples. Third, this study included no neurocognitive testing; therefore, we cannot analyse the relationship between brain structure abnormalities and behaviour patterns. Finally, the present study is cross-sectional in design. Without a longitudinal study, the differential effects of cancer progression, chemotherapy, and/or treatment-related factors cannot be determined. A longitudinal study with a larger sample would be valuable to further define the network properties of ALL patients.

In conclusion, our study demonstrated the presence of altered topological properties in the structural networks of ALL patients with chemotherapy. In addition, the network metrics suggested that the efficiency of the entire brain network was decreased. The abnormality of the structural networks between ALL patients with chemotherapy and healthy controls is remarkable and could contribute to a better understanding of the effects of chemotherapy in ALL patients.

## MATERIALS AND METHODS

### Participants

This study was approved by the Ethics committee of the Second Hospital of Anhui Medical University, and all participants obtained written informed consent. We identified 32 ALL patients who had completed all anti-cancer treatments with chemotherapy only in the Second Hospital of Anhui Medical University from June 2015 to June 2016. Four of the 32 survivors withdrew during data collection, leaving 28 participants (mean age: 40.71 ± 8.58 years; years since diagnosis: 7–38) included in the study. The ALL group had a history of chemotherapy treatment and was off-therapy for at least 3 years. Systemic chemotherapy included prednisolone, vincristine, cyclophosphamide, daunorubicin, doxorubicin, asparaginase, teniposide, cytarabine, 6-mercaptopurine, 6-thioguanine, and dexamethasone. Criteria for exclusion were as follows: (1) pre-existing neurologic disease affecting behaviour, (2) neurologic sequelae during treatment, (3) ALL relapse, (4) cranial irradiation or haematopoietic stem cell transplantation, and (5) MRI contraindication. For the control group, 20 healthy, age-matched controls were selected.

### MRI acquisition

All MRI examinations were performed with a 3.0 T MR scanner (Trio; Siemens, Erlangen, Germany) in the Second Hospital of Anhui Medical University. Foam pads and ear plugs were used to minimize head motion and scanner noise. Diffusion-weighted images were acquired with an echo planar imaging sequence covering the whole brain (TE/TR = 84/8400 ms, FOV = 256 × 256, slice thickness = 3 mm, slice gap = 0, b = 0,1000 s/mm^2^, direction = 30). High-resolution T1-weighted structural images were obtained with a magnetization-prepared rapid gradient-echo sequence (TE/TR = 2.98/1900 ms, FOV = 256 × 256, slice thickness = 1 mm, voxel size = 1 × 1 × 1 mm^3^, number of slices = 176).

### Data preprocessing

Data preprocessing and network construction were performed using PANDA (www.nitrc.org/projects/panda), a pipeline toolbox for diffusion MRI analysis [32]. Briefly, the image preprocessing steps included: format conversion of original data (DICOM), BET (skull removal), eddy current and head motion correction, fractional anisotropy (FA) calculation, and diffusion tensor tractography.

### Network construction

Defining network nodes. Individual structural images were first co-registered to their first b0 images. Next, the transformed structural images were non-linearly normalized to the MNI space. Finally, the derived deformation parameters were inverted and employed to warp the automated anatomical labelling (AAL) atlas [33] from the MNI space to the diffusion image native space. After this procedure, we obtained 90 cortical and subcortical regions (45 for each hemisphere), each representing a network node [23].

**Figure 3:**
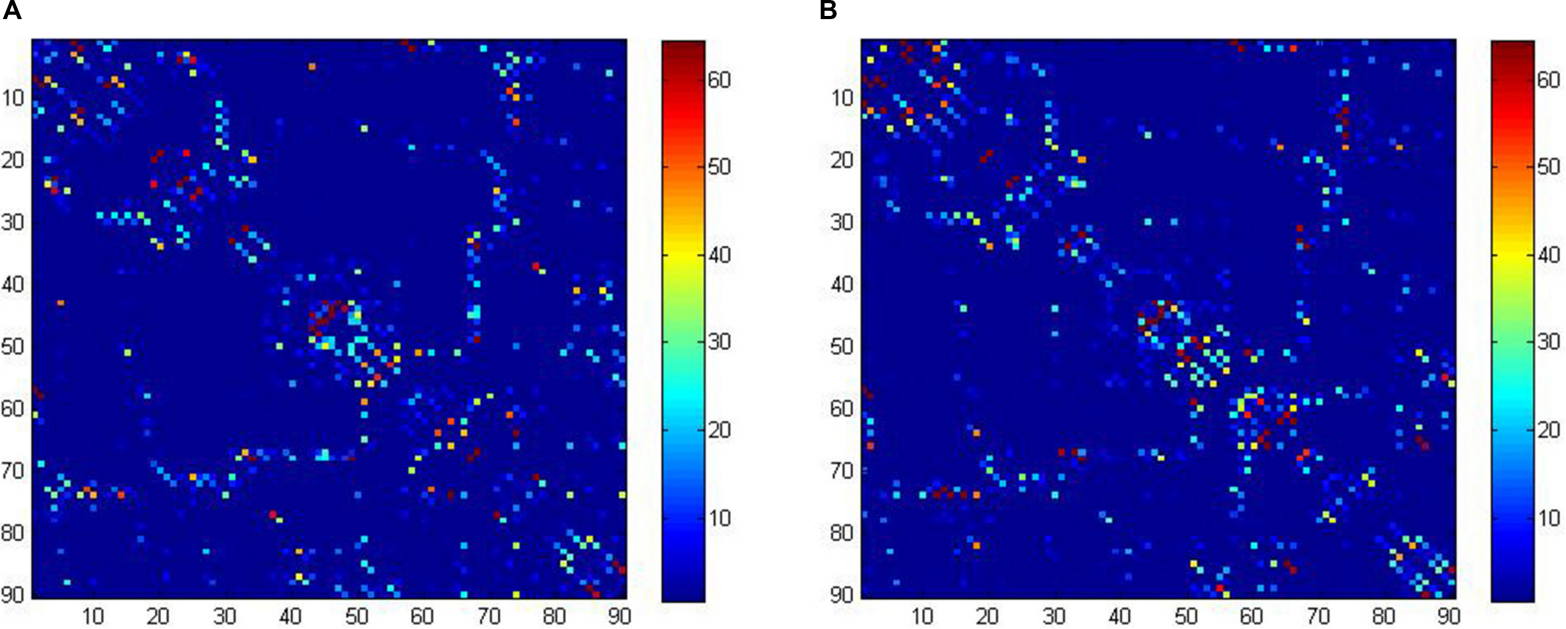
Structural connectivity (fiber number) matrices for the construction of structural weighted networks for ALL (**A**) and HC (**B**). The axes represent the 90 AAL brain regions used in the analysis. Color of each entry represents the level of connectivity. ALL, acute lymphoblastic leukemia with chemotherapy treatment, HC, healthy controls.

Defining network edges. To define the edges of the structural network, we calculated the number of fibres (with end-points in both nodes during the fibre tracking) between two regions, generating a 90 × 90 matrix for each subject (Figure 3). To reduce false-positive connections due to the limited resolution of DTI when a minimum of three fibres is used as a threshold [34–36], the fibre number (FN) between each couple of brain nodes was defined as the weights of the network edges. Finally, the FN-weighted structural networks were constructed for each participant [37, 38].

### Network analysis

The WM network topological properties were analysed using graph theory in GRETNA (www.nitrc.org/projects/gretna). For the structural-weighted brain network, we calculated the network metrics at the global level. We mainly focussed on the clustering coefficient Cp, shortest path length Lp, small-worldness (normalized clustering coefficient γ and normalized shortest path length λ) [26], local efficiency Eloc, and global efficiency Eg. The Cp of the entire network was the average of that of all nodes in the network, which refers to how the neighbouring nodes and the node itself are connected and indicates the local efficiency for the transformation of the information [23]. The Lp of the entire network is the average of that of all nodes in the network, which represents the average shortest travel distance between another node and the node itself and indicates the most efficient information transfer between the two nodes. To obtain a normalized clustering coefficient γ and a normalized shortest path length λ, we generated 100 matched random networks that had the same numbers of nodes and edges and degree distributions but preserved the weighted distribution of the real network. We calculated the average Cp_random_ and Lp_random_ over the random network. Then, we computed the γ (γ = Cp/Cp_random_) and λ (λ = Lp/Lp_random_), which indicate the normalized clustering coefficient and normalized shortest path length, respectively. In a small-world network, the γ should be much larger than 1, and the λ should be close to 1.

The global efficiency Eg is the average of the inverse of the shortest path length of all node pairs in the network and usually represents the capability of the network for performing parallel information processing. The local efficiency Eloc is the average of the global efficiency of the community neighbouring all nodes in the network and reflects the fault tolerance level of the network [39, 40].

### Identification of hubs

Hubs of structural networks are essential nodes that are identified in various ways [41]. In the present study, we applied betweenness to identify the hubs of the network. A node was considered as a hub if its regional betweenness centrality was one SD higher than the mean network betweenness.

### Statistical analysis

Statistical analysis was performed with SPSS 16.00 software. The group difference in age was tested using the two-sample *t*-test and the gender ratio was examined with a Pearson chi-square test. For group differences in global network measures (Cp, Lp, γ, λ, σ, Eloc, Eg), was used the two-sample *t*-test.

## SUPPLEMENTARY TABLE



## References

[1] Iyer NS, Balsamo LM, Bracken MB, Kadan-Lottick NS (2015). Chemotherapy-only treatment effects on long-term neurocognitive functioning in childhood ALL survivors: a review and meta-analysis. Blood.

[2] Kanellopoulos A, Andersson S, Zeller B, Tamnes CK, Fjell AM, Walhovd KB, Westlye LT, Fossa SD, Ruud E (2016). Neurocognitive Outcome in Very Long-Term Survivors of Childhood Acute Lymphoblastic Leukemia After Treatment with Chemotherapy Only. Pediatr Blood Cancer.

[3] Huang IC, Brinkman TM, Kenzik K, Gurney JG, Ness KK, Lanctot J, Shenkman E, Robison LL, Hudson MM, Krull KR (2013). Association between the prevalence of symptoms and health-related quality of life in adult survivors of childhood cancer: a report from the St Jude Lifetime Cohort study. J Clin Oncol.

[4] Gaynon PS, Angiolillo AL, Carroll WL, Nachman JB, Trigg ME, Sather HN, Hunger SP, Devidas M, Children's Oncology G (2010). Long-term results of the children's cancer group studies for childhood acute lymphoblastic leukemia 1983–2002: a Children's Oncology Group Report. Leukemia.

[5] Annett RD, Hile S, Bedrick E, Kunin-Batson AS, Krull KR, Embry L, MacLean WE, Noll RB (2015). Neuropsychological functioning of children treated for acute lymphoblastic leukemia: impact of whole brain radiation therapy. Psychooncology.

[6] Krull KR, Zhang N, Santucci A, Srivastava DK, Krasin MJ, Kun LE, Pui CH, Robison LL, Hudson MM, Armstrong GT (2013). Long-term decline in intelligence among adult survivors of childhood acute lymphoblastic leukemia treated with cranial radiation. Blood.

[7] Seigers R, Fardell JE (2011). Neurobiological basis of chemotherapy-induced cognitive impairment: a review of rodent research. Neurosci Biobehav Rev.

[8] Monje M, Dietrich J (2012). Cognitive side effects of cancer therapy demonstrate a functional role for adult neurogenesis. Behav Brain Res.

[9] Krull KR, Bhojwani D, Conklin HM, Pei D, Cheng C, Reddick WE, Sandlund JT, Pui CH (2013). Genetic mediators of neurocognitive outcomes in survivors of childhood acute lymphoblastic leukemia. J Clin Oncol.

[10] Aukema EJ, Caan MW, Oudhuis N, Majoie CB, Vos FM, Reneman L, Last BF, Grootenhuis MA, Schouten-van Meeteren AY (2009). White matter fractional anisotropy correlates with speed of processing and motor speed in young childhood cancer survivors. Int J Radiat Oncol Biol Phys.

[11] Schuitema I, Deprez S, Van Hecke W, Daams M, Uyttebroeck A, Sunaert S, Barkhof F, van Dulmen-den Broeder E, van der Pal HJ, van den Bos C, Veerman AJ, de Sonneville LM (2013). Accelerated aging, decreased white matter integrity, and associated neuropsychological dysfunction 25 years after pediatric lymphoid malignancies. J Clin Oncol.

[12] Edelmann MN, Krull KR, Liu W, Glass JO, Ji Q, Ogg RJ, Sabin ND, Srivastava DK, Robison LL, Hudson MM, Reddick WE (2014). Diffusion tensor imaging and neurocognition in survivors of childhood acute lymphoblastic leukaemia. Brain.

[13] Carey ME, Haut MW, Reminger SL, Hutter JJ, Theilmann R, Kaemingk KL (2008). Reduced frontal white matter volume in long-term childhood leukemia survivors: a voxel-based morphometry study. AJNR Am J Neuroradiol.

[14] Kesler SR, Tanaka H, Koovakkattu D (2010). Cognitive reserve and brain volumes in pediatric acute lymphoblastic leukemia. Brain Imaging Behav.

[15] Genschaft M, Huebner T, Plessow F, Ikonomidou VN, Abolmaali N, Krone F, Hoffmann A, Holfeld E, Vorwerk P, Kramm C, Gruhn B, Koustenis E, Hernaiz-Driever P (2013). Impact of chemotherapy for childhood leukemia on brain morphology and function. PLoS One.

[16] Kesler SR, Gugel M, Pritchard-Berman M, Lee C, Kutner E, Hosseini SM, Dahl G, Lacayo N (2014). Altered resting state functional connectivity in young survivors of acute lymphoblastic leukemia. Pediatr Blood Cancer.

[17] Robinson KE, Livesay KL, Campbell LK, Scaduto M, Cannistraci CJ, Anderson AW, Whitlock JA, Compas BE (2010). Working memory in survivors of childhood acute lymphocytic leukemia: functional neuroimaging analyses. Pediatr Blood Cancer.

[18] Edelmann MN, Ogg RJ, Scoggins MA, Brinkman TM, Sabin ND, Pui CH, Srivastava DK, Robison LL, Hudson MM, Krull KR (2013). Dexamethasone exposure and memory function in adult survivors of childhood acute lymphoblastic leukemia: A report from the SJLIFE cohort. Pediatr Blood Cancer.

[19] Luxton J, Brinkman TM, Kimberg C, Robison LL, Hudson MM, Krull KR (2014). Utility of the N-back task in survivors of childhood acute lymphoblastic leukemia. J Clin Exp Neuropsychol.

[20] Zeller B, Tamnes CK, Kanellopoulos A, Amlien IK, Andersson S, Due-Tonnessen P, Fjell AM, Walhovd KB, Westlye LT, Ruud E (2013). Reduced neuroanatomic volumes in long-term survivors of childhood acute lymphoblastic leukemia. J Clin Oncol.

[21] Tamnes CK, Zeller B, Amlien IK, Kanellopoulos A, Andersson S, Due-Tonnessen P, Ruud E, Walhovd KB, Fjell AM (2015). Cortical surface area and thickness in adult survivors of pediatric acute lymphoblastic leukemia. Pediatr Blood Cancer.

[22] Gong G, He Y, Concha L, Lebel C, Gross DW, Evans AC, Beaulieu C (2009). Mapping anatomical connectivity patterns of human cerebral cortex using in vivo diffusion tensor imaging tractography. Cereb Cortex.

[23] Bullmore E, Sporns O (2009). Complex brain networks: graph theoretical analysis of structural and functional systems. Nat Rev Neurosci.

[24] He Y, Evans A (2010). Graph theoretical modeling of brain connectivity. Curr Opin Neurol.

[25] Bullmore ET, Bassett DS (2011). Brain graphs: graphical models of the human brain connectome. Annu Rev Clin Psychol.

[26] Watts DJ, Strogatz SH (1998). Collective dynamics of ‘small-world’ networks. Nature.

[27] Hagmann P, Kurant M, Gigandet X, Thiran P, Wedeen VJ, Meuli R, Thiran JP (2007). Mapping human whole-brain structural networks with diffusion MRI. PloS one.

[28] Bassett DS, Bullmore E (2006). Small-world brain networks. Neuroscientist.

[29] He Y, Chen ZJ, Evans AC (2007). Small-world anatomical networks in the human brain revealed by cortical thickness from MRI. Cereb Cortex.

[30] Liu F, Zhuo C, Yu C (2016). Altered Cerebral Blood Flow Covariance Network in Schizophrenia. Front Neurosci.

[31] Rubinov M, Sporns O (2010). Complex network measures of brain connectivity: uses and interpretations. Neuroimage.

[32] Kesler SR, Gugel M, Huston-Warren E, Watson C (2016). Atypical Structural Connectome Organization and Cognitive Impairment in Young Survivors of Acute Lymphoblastic Leukemia. Brain Connect.

[33] Latora V, Marchiori M (2001). Efficient behavior of small-world networks. Phys Rev Lett.

[34] Zhong Z, Zhao T, Luo J, Guo Z, Guo M, Li P, Sun J, He Y, Li Z (2014). Abnormal topological organization in white matter structural networks revealed by diffusion tensor tractography in unmedicated patients with obsessive-compulsive disorder. Prog Neuropsychopharmacol Biol Psychiatry.

[35] Zou L, Su L, Xu J, Xiang L, Wang L, Zhai Z, Zheng S (2017). Structural brain alteration in survivors of acute lymphoblastic leukemia with chemotherapy treatment: A voxel-based morphometry and diffusion tensor imaging study. Brain Res.

[36] Sporns O, Honey CJ, Kotter R (2007). Identification and classification of hubs in brain networks. PloS one.

[37] Cui Z, Zhong S, Xu P, He Y, Gong G (2013). PANDA: a pipeline toolbox for analyzing brain diffusion images. Front Hum Neurosci.

[38] Tzourio-Mazoyer N, Landeau B, Papathanassiou D, Crivello F, Etard O, Delcroix N, Mazoyer B, Joliot M (2002). Automated anatomical labeling of activations in SPM using a macroscopic anatomical parcellation of the MNI MRI single-subject brain. Neuroimage.

[39] Li Y, Liu Y, Li J, Qin W, Li K, Yu C, Jiang T (2009). Brain anatomical network and intelligence. PLoS Comput Biol.

[40] Shu N, Liu Y, Li K, Duan Y, Wang J, Yu C, Dong H, Ye J, He Y (2011). Diffusion tensor tractography reveals disrupted topological efficiency in white matter structural networks in multiple sclerosis. Cereb Cortex.

[41] Zhu J, Wang C, Liu F, Qin W, Li J, Zhuo C (2016). Alterations of Functional and Structural Networks in Schizophrenia Patients with Auditory Verbal Hallucinations. Front Hum Neurosci.

[42] Baggio HC, Segura B, Junque C, de Reus MA, Sala-Llonch R, Van den Heuvel MP (2015). Rich Club Organization and Cognitive Performance in Healthy Older Participants. J Cogn Neurosci.

[43] Zhao T, Cao M, Niu H, Zuo XN, Evans A, He Y, Dong Q, Shu N (2015). Age-related changes in the topological organization of the white matter structural connectome across the human lifespan. Hum Brain Mapp.

[44] Achard S, Bullmore E (2007). Efficiency and cost of economical brain functional networks. PLoS Comput Biol.

