# Correction: Abnormal topological organization in white matter structural networks in survivors of acute lymphoblastic leukaemia with chemotherapy treatment

**DOI:** 10.18632/oncotarget.28535

**Published:** 2023-12-12

**Authors:** Liwei Zou, Lianzi Su, Rongmiao Qi, Fang Bao, Xianjing Fang, Longsheng Wang, Zhimin Zhai, Dan Li, Suisheng Zheng

**Affiliations:** ^1^Department of Radiology, The Second Hospital of Anhui Medical University, Anhui, China; ^2^Department of Hematology, The Second Hospital of Anhui Medical University, Anhui, China; ^3^Department of Scientific Research, The Second Hospital of Anhui Medical University, Anhui, China; ^4^Medical Image Research Center, Anhui Medical University, Anhui, China; ^*^Co-first authors

**This article has been corrected:** In the Materials and Methods section, under the ‘MRI Acquisition,’ heading, the model of MRI scanner has been incorrectly spelled as “All MRI examinations were performed with a 3.0 T MR scanner (Trio; Siemens, Erlangen, German) in the Second Hospital of Anhui Medical University.” However, the correct model is “All MRI examinations were performed with a 3.0 T MR scanner (Verio; Siemens, Erlangen, German) in the Second Hospital of Anhui Medical University.” The authors declare that these corrections do not change the results or conclusions of this paper.

Original article: Oncotarget. 2017; 8:60568–60575. 60568-60575. https://doi.org/10.18632/oncotarget.19104

