# Supplementary material for: Abnormal topological organization in white matter structural networks in survivors of acute lymphoblastic leukaemia with chemotherapy treatment
Source: Oncotarget. 2017 Jul 8;8(36):60568–75. doi: 10.18632/oncotarget.19104 (PMC5601162; doi:10.18632/oncotarget.19104)
Supplement: Supplementary file 1 [file oncotarget-08-60568-s001.pdf]

# Abnormal topological organization in white matter structural networks in survivors of acute lymphoblastic leukaemia with chemotherapy treatment

## SUPPLEMENTARY MATERIALS

### Supplementary Table 1: The effect of age on the global network efficiency were analyzed by GLM

**Tests of Between-Subjects Effects**

**Dependent Variable:Eg**

| Source | Type III Sum of Squares | df | Mean Square | F | Sig. |
|---|---|---|---|---|---|
| Corrected Model | 4.291[a] | 2 | 2.145 | .374 | .690 |
| Intercept | 84.988 | 1 | 84.988 | 14.832 | .000 |
| age | 1.450 | 1 | 1.450 | .253 | .617 |
| group | 1.960 | 1 | 1.960 | .342 | .562 |
| Error | 257.857 | 45 | 5.730 | | |
| Total | 2446.068 | 48 | | | |
| Corrected Total | 262.148 | 47 | | | |

a. R Squared = .016 (Adjusted R Squared = −.027).
